# Parents’ empathic perspective taking and altruistic behavior predicts infants’ arousal to others’ emotions

**DOI:** 10.3389/fpsyg.2015.00360

**Published:** 2015-04-02

**Authors:** Michaela B. Upshaw, Cheryl R. Kaiser, Jessica A. Sommerville

**Affiliations:** ^1^Early Childhood Cognition Lab, Department of Psychology, Center for Child and Family Well-being, University of WashingtonSeattle, WA, USA; ^2^Social Identity Lab, Department of Psychology, University of WashingtonSeattle, WA, USA

**Keywords:** infancy, pupil dilation, empathy, arousal, parental dispositions

## Abstract

Empathy emerges in children’s overt behavior around the middle of the second year of life. Younger infants, however, exhibit arousal in response to others’ emotional displays, which is considered to be a precursor to fully developed empathy. The goal of the present study was to investigate individual variability in infants’ arousal toward others’ emotional displays, as indexed by 12- and 15-month-old infants’ (*n* = 49) pupillary changes in response to another infant’s emotions, and to determine whether such variability is linked to parental empathy and prosociality, as indexed via self-report questionnaires. We found that increases in infants’ pupil dilation in response to others’ emotional displays were associated with aspects of parental empathy and prosociality. Specifically, infants who exhibited the greatest arousal in response to others’ emotions had parents who scored highly on empathic perspective taking and self-reported altruism. These relations may have been found because arousal toward others’ emotions shares certain characteristics with empathic and prosocial dispositions. Together, these results demonstrate the presence of early variability in a precursor to mature empathic responding in infancy, which is meaningfully linked to parents’ empathic dispositions and prosocial behaviors.

## Introduction

Empathy, or the understanding and experiencing of another’s affective or psychological state, is integral to fostering positive social interactions and healthy interpersonal relationships. Indeed, empathy is considered to be vital to the emergence of prosocial behaviors (Hoffman, 1982; de Waal, 2008; Knafo et al., 2008). This theoretical claim is bolstered by empirical work demonstrating that higher levels of empathy in both children and adults are associated with an increased likelihood of offering help to a stranger (Dovidio et al., 1990), donating money to charity (Miller, 1979; Davis, 1983), and an increased willingness to encounter and help needy individuals (e.g., volunteering at a shelter; Davis et al., 1999; Davis, 2005; see also Eisenberg and Miller, 1987; Batson, 1991, 2002 for reviews). A critical question, then, concerns the developmental origins of empathy, as well as when in ontogeny individual differences emerge in empathic responses. The goal of this paper is to investigate an early precursor to empathy, particularly infants’ arousal in response to others’ emotions, and to examine whether variability in such arousal is associated with parental dispositions, such as empathy, and theoretically aligned characteristics, such as prosocial behavior.

In its mature form, empathy is considered to have both affective and cognitive components (Davis, 1983; Zahn-Waxler and Radke-Yarrow, 1990; Eisenberg, 2000; Preston and de Waal, 2002; Knafo et al., 2008). The cognitive component of empathy involves apprehending or understanding another person’s experience and differentiating that from one’s own (i.e., putting oneself in another person’s ‘shoes’; Davis, 1983; Zahn-Waxler and Radke-Yarrow, 1990). The affective component of empathy involves one’s emotional response toward another person’s experience (i.e., feelings of warmth, compassion, and concern toward others; Davis, 1983; Batson et al., 1987; Zahn-Waxler and Radke-Yarrow, 1990; Eisenberg, 2000). Previous work has found evidence for both cognitive and affective components of empathy by 18 months of age: for example, toddlers attempt to actively comfort an upset experimenter and actively seek information regarding the source of the experimenter’s distress (Zahn-Waxler et al., 1979, 1992b). Importantly, past work also reveals that there is variability in young children’s empathic responses by the second year of life (Zahn-Waxler et al., 1979, 1992b; Eisenberg et al., 1989). This individual variability in early empathic responding appears to be stable across contexts (Young et al., 1999; Robinson et al., 2001; Spinrad and Stifter, 2006; Moreno et al., 2008) and time (Zahn-Waxler et al., 1992a,b, 2001; Volbrecht et al., 2007). Nevertheless, because many previous paradigms (e.g., Zahn-Waxler et al., 1992a,b) have relied on children’s overt verbal and behavioral responses to another person’s distress, existing work may overestimate the age at which children first begin to demonstrate variability in empathic responses. This raises the possibility that individual differences in precursors to empathy may be present even earlier in development.

Indeed, well before overt signs of empathy emerge in development, infants exhibit arousal in response to others’ emotional expressions. For example, newborns cry in response to hearing another infant’s cry, but not in response to their own cry or to sounds with matched synthetic frequencies (Sagi and Hoffman, 1976; Martin and Clark, 1982; Dondi et al., 1999). Between 2 and 3 months of age, infants change their affective state in response to their mother’s emotional expressions (e.g., angry facial expressions and freezing in response to their mother’s expression of anger; Haviland and Lelwica, 1987), and by about 9 months of age, infants tailor their behavior to correspond with their mother’s emotions, such as playing and smiling more frequently when their mother exhibits joy as opposed to sadness (Termine and Izard, 1988). These behaviors are considered to be precursors to more mature empathic responding because, in order to respond empathically, one must first register and be moved by another’s emotional expression (Hoffman, 1982, 2000, 2008). Critically, infants’ ability to register others’ emotions has been linked to the later emergence of empathic behaviors (Roth-Hanania et al., 2011). For example, infants’ facial and vocal signs of concern in response to their mother’s distress at 10 months is positively associated with their attempts to help and comfort their mother during simulated expressions of pain at 12–16 months of age. Altogether, this work motivates a closer investigation into precursors to later empathic responses.

In order to track the development of empathy and related precursors, researchers are increasingly turning to physiological measures. One particularly promising physiological measure is pupil dilation, or changes in pupil size that occur during stimulus processing. Pupil dilation reflects activity of the sympathetic nervous system and is thought to index increased attention and arousal in response to the observed stimuli (Beatty and Lucero-Wagoner, 2000; Porter et al., 2007; Laeng et al., 2012). Of central importance to the present study, pupil dilation has been used to measure arousal in response to others’ emotions (Partala and Surakka, 2003; Bradley et al., 2008; Geangu et al., 2011b). For example, infants and adults exhibit greater pupil dilation during the processing of emotional stimuli (i.e., stimuli with a positive or negative valence) relative to the processing of neutral stimuli (Partala and Surakka, 2003; Bradley et al., 2008). In addition, this work has shown that the processing of negative emotional stimuli leads to greater pupil dilation than the processing of positive emotional stimuli (e.g., Geangu et al., 2011b). Thus, changes in pupil size, and in particular, pupil dilation, reflect one’s degree of arousal in response to others’ emotional displays. Pupil dilation is ideal for investigating arousal in response to others’ emotions in infancy, as pupil dilation is an automatically elicited, non-verbal response, that does not require infants to produce complex, overt behavior, nor possess a sophisticated understanding of the observed situation (Laeng et al., 2012). In addition, pupil dilation in response to others’ emotions is variable across individuals, suggesting that this measure is well suited to capture individual differences or variability in arousal toward others’ emotions (Vanderhasselt et al., 2014; see also Bitsios et al., 2004).

Past research has investigated factors that contribute to the development of empathy in childhood, most notably investigating the impact of parental behaviors. Perhaps unsurprisingly, this work has largely focused on parental behaviors in the context of parent–child interactions, and has generally found that parents who exhibit sensitivity and concern in response to their children’s distress are more likely to have children who exhibit greater empathic responding toward others (Zhou et al., 2002; Strayer and Roberts, 2004; Taylor et al., 2013; Newton et al., 2014). In contrast, the impact of parental dispositions, broadly construed, such as the parents’ personality and their tendencies to engage in certain behaviors, on children’s empathy has been less well studied. Nevertheless, existing research has demonstrated relations between parents’ dispositional empathy and children’s empathy. For example, parents who report feeling more empathic concern toward others in their everyday lives (e.g., endorsing statements such as, “When I see someone being taken advantage of, I feel kind of protective toward them”) have children who exhibit more empathic behaviors toward others in need (e.g., child tries to comfort or reassure another in distress; Eisenberg et al., 1991; Volling et al., 2008). In addition, mothers who score high on measures of dispositional empathy and low on measures of personal distress in response to others’ misfortunes have children who exhibit more empathic concern toward needy others as well as an enhanced capacity to adopt others’ perspectives (Davidov and Grusec, 2006). Thus, how parents think, feel, and act toward other people influences the development of children’s empathy, even when such thoughts, feelings, and actions occur outside of the parent–child relationship. This association between parents and children could be due to genetic similarities, or to parental dispositions influencing their everyday behavior, of which children are frequently exposed to. Regardless, the relation between parents’ empathic dispositions and related behaviors and children’s developing empathy warrants closer investigation.

In the present study, we used pupil dilation to index infants’ arousal in response to others’ emotions and investigated whether individual variability in infants’ pupillary changes in response to others’ emotional displays was related to parents’ empathic dispositions and prosocial tendencies. Prior work investigating infants’ pupillary responses toward other infants’ emotions has demonstrated group level increases in infants’ pupil diameter in response to happy and sad emotional expressions relative to pupil diameter during neutral emotional expressions (Geangu et al., 2011b). Thus, in the current study, infants between 12- and 15-months of age watched videos of other infants expressing happiness and sadness, as well as neutral emotionality, while changes in their pupil diameter were recorded using an eye-tracker. In order to assess parents’ empathic dispositions and prosocial tendencies, infants’ primary caregiver completed two, widely used questionnaires that measure self-reported dispositional empathy and prosociality: the Interpersonal Reactivity Index (IRI; Davis, 1983) and the Prosocial Personality Battery (PSB; Penner et al., 1995), respectively. We predicted that parents who report greater levels of dispositional empathy, and who report a higher frequency of performing helpful behaviors toward others, would have infants who exhibit greater arousal, as assessed via changes in pupil diameter, during observation of another infant’s emotional displays.

## Materials and Methods

### Participants

The final sample included 22 (*n* = 13 female), 12-month-olds (*M* = 12 months and 5 days; range: 11 months and 23 days to 12 months and 16 days) and 27 (*n* = 14 female), 15-month-olds (*M* = 15 months and 12 days; range: 14 months and 25 days to 16 months and 10 days), who were recruited from a database maintained by a large university in the Pacific Northwest of the United States. Thirteen additional infants participated but were excluded from analysis due to insufficient data stemming from technical problems with the eye-tracking camera and software (*n* = 8) or because of fussiness (*n* = 5). Of these infants, 41 were Caucasian, one was Native American, one was Hispanic, nine were of mixed ethnic backgrounds, and two parents chose not to disclose this information. Before participating, all parents provided informed consent for their infants and themselves to participate in the study.

### Stimuli

The video stimuli were adapted from Geangu et al. (2011b; see article for full details and description of the original stimuli) and were presented using Presentation®; software (Version 0.70, www.neurobs.com). In the neutral video, a male infant displayed neutral facial expressions and produced neutral babbling vocalizations (without emotional prosody). In the happy video, a different male infant displayed happy facial expressions and produced laughing vocalizations. In the sad video, a third male infant displayed facial expressions of sadness and frustration and produced strong crying vocalizations. Each video was 25 s in length (reduced from 50 s, as prior work found that infants’ attention wandered during the second half of the video; Geangu et al., 2011b). In shortening the video length, care was taken to select segments of the original video that contained the least amount of infant movement, in order to reduce luminance differences. As an additional control for luminance differences, the videos were presented in black and white. Lastly, the videos were cropped to reduce the amount of background imagery and to enhance focus on the infants’ emotional expressions. After these adaptations, we extracted the spatial average of the RGB values for each frame of each video, and calculated the weighted sum of the RGB values to estimate photometric luminance for each video (i.e., luminance = (0.2126 ^∗^ R) + (0.7152 ^∗^ G) + (0.0722 ^∗^ B); see Jackson and Sirois, 2009). This analysis confirmed that the videos did not differ in photometric luminance: 8.65 = neutral, 8.66 = happy, and 8.36 = sad (all comparisons *ns*).

Infants were also shown a 10 s baseline video which consisted of a red and white rattle moving back-and-forth against a black background accompanied by soft music. The baseline video served to break up and transition infants’ attention between the emotional videos. In addition, the baseline video provided a baseline assessment of infants’ pupil size, which was used to perform baseline corrections prior to data analysis. We used the same baseline video as in Geangu et al. (2011b) in order to aid comparability between the two studies.

### Apparatus

Infants’ pupil diameter was measured using an Applied Science Laboratories (ASL) Eye-Trac 6 Control Unit and Desktop Optics D6 camera; accuracy 0.5°, resolution 0.26°, and collected at a frequency of 60 Hz. The eye-tracking camera was positioned beneath the stimulus displaying monitor (measuring 68.6 cm diagonally), and both the camera and monitor were placed directly in front of a plain beige wall. Dark curtains surrounded the stimulus displaying monitor and the infant, in order to focus infants’ attention, and no other stimuli were present that could distract infants’ attention. The lighting in the experimental room was held constant across participants, in order to prevent pupil size changes as a function of ambient lighting differences.

### Procedure

All study procedures were approved by the university’s Internal Review Board before the research was conducted. Infants sat in a car seat, approximately 76.2 cm from the stimulus displaying monitor, and the infant’s parent sat behind them, out of the infant’s sight. After the infant was in position, and before pupil data were recorded, a five-point calibration was performed (see Gredebäck et al., 2010). For approximately half of participants (*n* = 22), the procedure began with the presentation of the neutral video, followed by the happy video. For the other half of participants (*n* = 27), the procedure began with the presentation of the happy video, followed by the neutral video. Infants were always shown the sad video last in the series of three videos, as prior work has found that negative stimuli can have carryover effects (Geangu et al., 2011a,b). Before each emotional video, infants were shown the 10 s baseline video.

### Data Processing

Infants’ pupil diameter was filtered off-line using Matlab (version 7.11 0.584, R2010b, Natick, MA, USA). A 20-point moving average window was applied to the data (i.e., pupil diameter at each time point was calculated as the average diameter of the surrounding 20 data points) in order to remove sudden brief increases and decreases in pupil diameter that normally occur and are considered to be artifacts (Beatty and Lucero-Wagoner, 2000; Geangu et al., 2011b). For analysis purposes, pupil diameter was calculated as the average pupil diameter during the last 23 s of each video. Infants’ pupil diameter during the first 2 s of each video was excluded from analysis because of pupillary reflexes related to the baseline video to stimulus transition. Before data analysis, infants’ pupil diameter was baseline-corrected by subtracting the average pupil diameter during the last second of the preceding baseline video from the average pupil diameter during (the last 23 s of) each emotional video. Using a baseline-corrected measure of pupil diameter controls for differences in tonic pupil size, as well as circumvents ‘drift’ in pupillary size during the task, which can occur due to the emotional nature of the stimuli (Hess and Polt, 1960; Geangu et al., 2011b).

### Parental Questionnaire Measures

Before participating in the study, infants’ primary caregivers completed the IRI (Davis, 1983) and the PSB (Penner et al., 1995). Among the 47 primary caregivers who completed the questionnaires, *n* = 43 were mothers of the infant participants, and *n* = 4 were fathers of the infant participants. Scores on the IRI were exclusively used to investigate relations with parents’ dispositional empathy, as the IRI is the most widely used assessment of dispositional empathy (e.g., Beven et al., 2004; Pulos et al., 2004; Koller and Lamm, 2014), and because there is considerable overlap between items assessing dispositional empathy on the PSB and the IRI. Scores on the PSB were used to assess parents’ prosocial behavioral tendencies.

The IRI is composed of four subscales (seven items for each subscale) that assess cognitive and affective components of dispositional empathy. Two subscales that assess affective aspects of empathy (empathic concern and personal distress) and one subscale that assesses the cognitive aspect of empathy (perspective taking) were analyzed for the present study. Empathic concern measures the tendency for one to experience other-oriented feelings of empathy and compassion for less fortunate individuals (e.g., “I often have tender, concerned feelings for people less fortunate than me”). In contrast to empathic concern, personal distress measures the tendency to experience *self-*oriented feelings of distress during others’ misfortunes (e.g., “When I see someone who badly needs help in an emergency, I go to pieces”). Thus, personal distress is thought to impede one’s ability to behave empathically during others’ misfortunes, as one must first overcome self-oriented feelings of anxiety and discomfort. On the cognitive side of empathy, the perspective taking subscale measures the tendency for one to adopt the point-of-view of another person when appraising a social situation (e.g., “I try to look at everybody’s side of a disagreement before I make a decision”). Items on the IRI were rated on a five-point scale ranging from 1 (*does not describe me well*) to 5 (*describes me very well*). Internal consistency (Cronbach’s alpha) was 0.75 for the empathic concern subscale, 0.82 for the perspective taking subscale, and 0.79 for the personal distress subscale.

The PSB is a self-report measure of dispositional empathy and the frequency of performing prosocial and helpful behaviors toward others. Scores on subscales of the PSB are combined in order to represent two higher-order factors: Other-Oriented Empathy, which measures the tendency to feel concern, pity, or sorrow in response to others’ distress (e.g., “I am often quite touched by things that I see happen.”) and Helpfulness, which measures the tendency for one to engage in helpful and altruistic behaviors toward others (e.g., “I have helped carry a stranger’s belongings.”). The higher-order factor of Helpfulness is constructed from scores on two subscales: self-reported altruism (composed of five items) and personal distress (composed of three items). However, because the items used to assess personal distress on the PSB are a verbatim subset of the items used to assess personal distress on the IRI, only parental scores on the self-reported altruism subscale of the PSB were used in the present analysis. Items on the self-reported altruism subscale of the PSB were rated on a five-point scale ranging from 1 (*never*) to 5 (*very often*). Internal consistency (Cronbach’s alpha) was 0.74 for the self-reported altruism subscale.

## Results

### Infants’ Visual Attention to the Emotional Videos

Our first course of action was to ensure that infants visually attended to the emotional videos. Accordingly, we calculated the percentage of time infants spent looking toward (versus away from) each emotional video relative to the total video duration. We conducted a 3 × 2 ANOVA with emotion (happy, neutral, sad) as a within-subjects factor and infants’ age (12 or 15 months) as a between-subjects factor on the percentage of time infants spent looking toward the emotional videos. We found a significant main effect of emotion, *F*(2,94) = 5.88, *p* = 0.004, $\eta_{p}^{2}$ = 0.11, and no other main effects or interactions. Accordingly, we collapsed across infant age in order to further explore the main effect of emotion. Paired samples *t*-tests reveal that infants spent significantly more time looking toward the sad video (*M* = 76.6% of the total video duration, *SE* = 3.4%) relative to the happy (*M* = 67.2%, *SE* = 3.3%), *t*(48) = 2.32, *p* = 0.03, *d* = 0.50, and neutral videos, (*M* = 64.2%, *SE* = 3.6%), *t*(48) = 3.25, *p* = 0.002, *d* = 0.40. There was no difference in the amount of time infants looked toward the happy and neutral videos, *t*(48) = -0.99, *p* = 0.33.

### Infants’ Pupil Diameter in Response to the Emotional Videos

**FIGURE 1:**
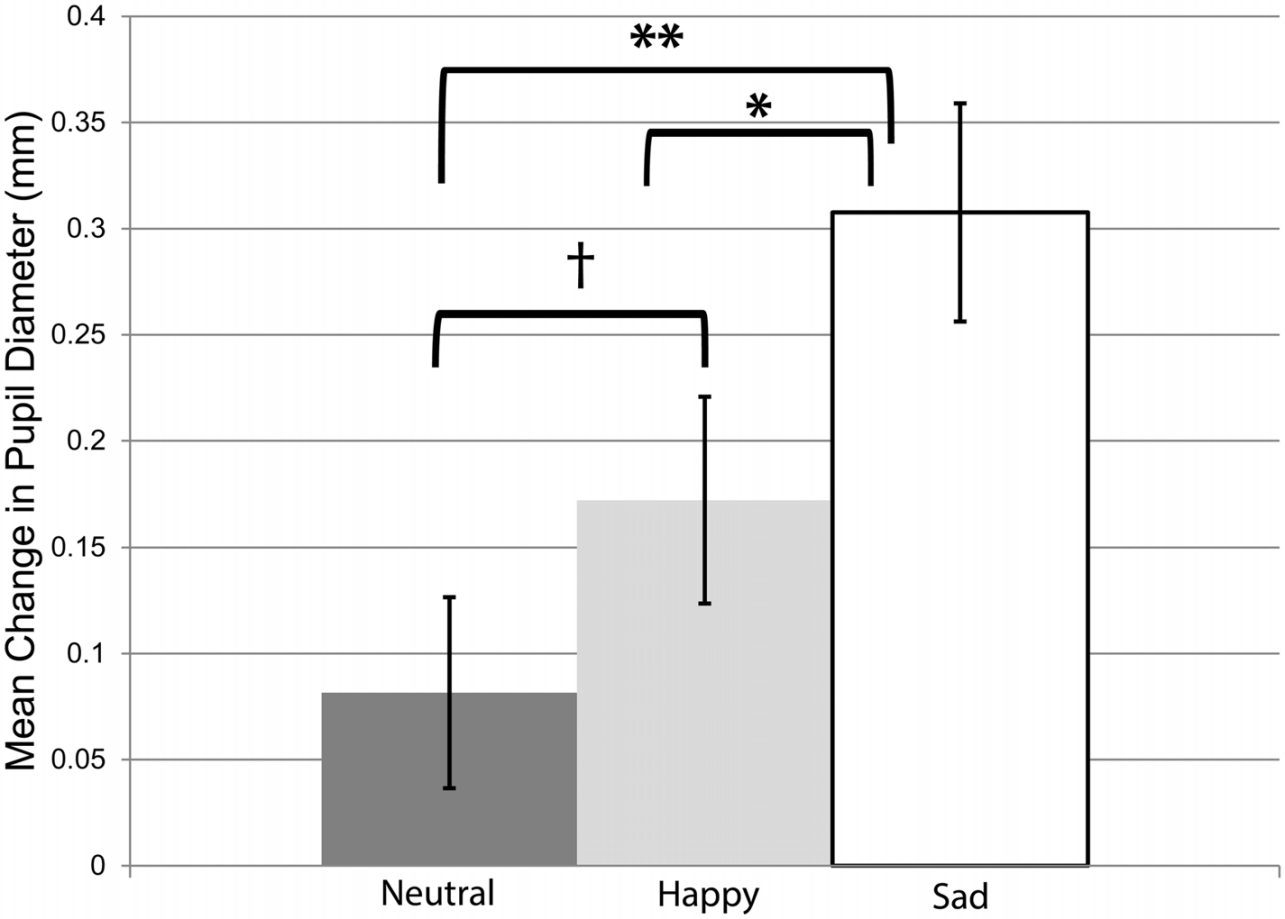
**Mean change in infants’ pupil diameter during observation of the emotional videos (relative to pupil diameter during observation of the baseline video; in mm)**. Error bars represent *SE*. ^†^*p* = 0.08, ^∗^*p* = 0.01, ^∗∗^*p* < 0.01.

Our next course of action was to ensure that the videos elicited infants’ arousal in response to others’ emotional displays, in order to validate our paradigm and to replicate prior work (Geangu et al., 2011b). Thus, we conducted a 3 × 2 ANOVA, with emotion (happy, neutral, sad) as a within-subjects factor, and infants’ age (12 or 15 months) and stimulus order (happy video first or neutral video first) as between-subjects factors, on infants’ pupil diameter during observation of the emotional videos. We found a main effect of emotion, *F*(2,90) = 8.20, *p* = 0.001, $\eta_{p}^{2}$ = 0.14, and no other main effects or interactions. Accordingly, we collapsed across infant age and stimulus order in subsequent analyses on infants’ pupil dilation during the emotional videos. Planned, paired *t*-tests to investigate the main effect of emotion confirm that greater pupil dilation was found during observation of the sad video (*M* = 0.31 mm, *SE* = 0.05) relative to the neutral video (*M* = 0.08 mm, *SE* = 0.04), *t*(48) = 3.99, *p* < 0.01, *d* = 0.48, and relative to the happy video (*M* = 0.17 mm, *SE* = 0.05), *t*(48) = 2.60, *p* = 0.01, *d* = 0.37. In addition, pupil dilation during the happy video was marginally greater than pupil dilation during the neutral video, *t*(48) = 1.77, *p* = 0.08, *d* = 0.25 (see **Figure 1**). Importantly, the amount of time that infants spent looking toward the emotional videos was unrelated to their degree of pupil dilation in response to the videos, as assessed by Pearson’s correlations between infants’ percentage of looking toward each video and their pupil diameter in response to it: happy, *r*(49) = -0.23, *p* = 0.12; neutral, *r*(49) = -0.17, *p* = 0.26; sad, *r*(49) = -0.04, *p* = 0.76.

### Parental Questionnaire Measures: Descriptive Statistics

As a result of skipped questionnaire items and/or un-readable questionnaire responses, scores are missing from: three parents for the empathic concern subscale of the IRI and the self-reported altruism subscale of the PSB, two parents for the perspective taking subscale of the IRI, and four parents for the personal distress subscale of the IRI. Two parents did not complete any of the questionnaire measures. Means, *SE*s, and ranges for each of the subscales are presented in **Table 1**.

**Table 1:** Pearson’s correlations, means, SEs, and ranges for the main study variables.

Variable	1	2	3	4	5	6	7
1. Perspective taking (IRI)	__						
2. Empathic concern (IRI)	0.49^∗∗^	__					
3. Personal distress (IRI)	-0.32^∗^	-0.14	__				
4. Self-reported altruism (PSB)	0.13	0.01	-0.19	__			
5. Composite pupil dilation difference scores^a^	0.34^∗^	-0.02	-0.26^†^	0.32^∗^	__		
6. Happy pupil dilation difference scores^a^	0.28^†^	-0.01	-0.24	0.37^∗^	0.86^∗∗^	__	
7. Sad pupil dilation difference scores^a^	0.31^∗^	-0.02	-0.21	0.20	0.89^∗∗^	0.54^∗∗^	__

Mean	18.96	20.70	9.62	15.24	0.32	0.09	0.23
*SE*	0.64	0.64	0.75	0.54	0.09	0.05	0.06
Possible range	0–28	0–28	0–28	5–25	–	–	–
Actual range	7–27	6–28	1–21	8–25	-1.01–2.09	-0.61–1.13	-0.56–1.35

*^a^Pearson’s correlations are controlling for infants’ age*.

*^∗∗^*p* < 0.001, ^∗^*p* < 0.05, ^†^*p* = 0.08*.

As shown in **Table 1**, scores on the perspective taking subscale were significantly associated with scores on the empathic concern subscale, *r*(46) = 0.49, *p* < 0.001, as well as significantly negatively associated with scores on the personal distress subscale, *r*(47) = -0.32, *p* = 0.03. However, scores on the empathic concern subscale were unrelated to scores on personal distress, *r*(46) = -0.14, *p* = 0.37. Parental scores on the self-reported altruism subscale of the PSB were unrelated to scores on the empathic concern subscale of the IRI, *r*(45) = 0.01, *p* = 0.94, the perspective taking subscale of the IRI, *r*(46) = 0.13, *p* = 0.38, and the personal distress subscale of the IRI, *r*(46) = -0.19, *p* = 0.20.

### Relations Between Infants’ Pupil Dilation in Response to Others’ Emotions and Parental Questionnaire Measures

In order to capture changes in infants’ pupil dilation during the sad and happy emotional videos relative to their pupil dilation during the neutral video, we created two difference scores by subtracting infants’ pupil diameter during the neutral video from their pupil diameter during the sad and happy videos (hereafter referred to as “sad pupil dilation difference scores” and “happy pupil dilation difference scores”). These scores were designed to capture the difference in infants’ pupil dilation during the sad (or happy) videos relative to their pupil dilation during the neutral videos. To capitalize on changes in infants’ pupil dilation in response to *both* of the happy and sad emotional displays, we computed a composite pupil dilation difference score by summing the sad and happy pupil dilation difference scores together. In the following analyses, we first conducted Pearson’s correlations between infants’ composite pupil dilation difference scores (controlling for infants’ age) and parents’ scores on the questionnaire measures (empathic concern, perspective taking, and personal distress subscales of the IRI, and the self-reported altruism subscale of the PSB). If this correlation was significant, we then conducted separate correlations between infants’ happy and sad pupil dilation difference scores and the parental variable of interest.

As shown in **Table 1**, parental scores on the perspective taking subscale were significantly associated with infants’ composite pupil dilation difference scores, *r*(44) = 0.34, *p* = 0.02, 95% CI [0.05, 0.58], such that higher parental perspective taking predicted greater pupil dilation during the happy and sad videos relative to the neutral video. In addition, parental scores on the personal distress subscale were marginally negatively associated with infants’ composite pupil dilation difference scores, *r*(44) = -0.26, *p* = 0.08, indicating that parents who report experiencing less feelings of self-oriented distress during others’ misfortunes have infants who exhibit more pupil dilation in response to others’ emotional displays. However, no relation was found between parents’ scores on the empathic concern subscale and infants’ composite pupil dilation difference scores, *r*(43) = -0.02, *p* = 0.92. Lastly, we found a significant relation between parental scores on the self-reported altruism subscale of the PSB and infants’ composite pupil dilation difference scores, *r*(43) = 0.32, *p* = 0.03, 95% CI [0.02, 0.57], suggesting that parents who report performing more frequent altruistic behavior have infants who exhibit greater pupil dilation in response to others’ emotions.

**FIGURE 2:**
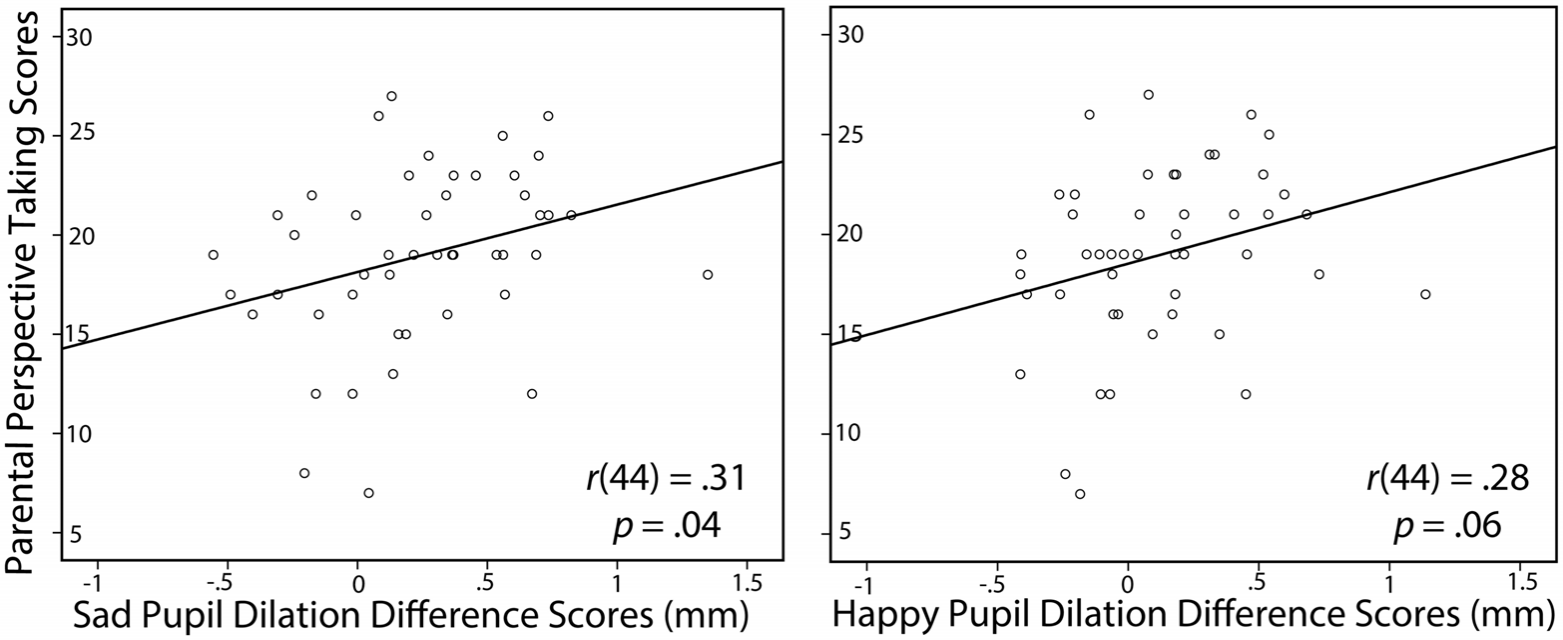
**Pearson’s correlations (controlling for infants’ age) between parental perspective taking scores (IRI) and infants’ sad and happy pupil dilation difference scores (in mm)**.

**FIGURE 3:**
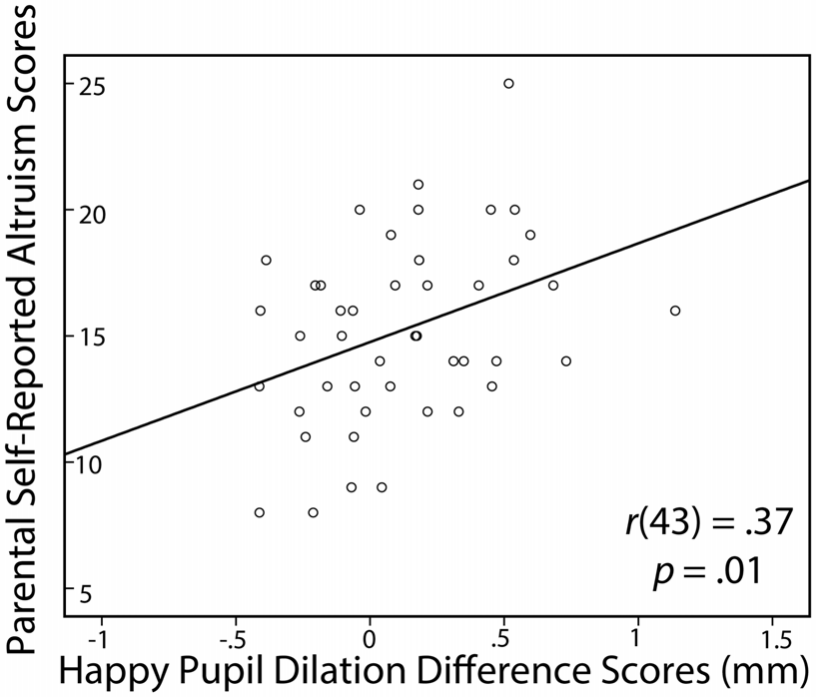
**Pearson’s correlations (controlling for infants’ age) between parental self-reported altruism scores (PSB) and infants’ happy pupil dilation difference scores (in mm)**.

In order to further examine the significant relations between parental scores on the perspective taking and self-reported altruism subscales and infants’ composite pupil dilation difference scores, we conducted correlations between parental scores on the perspective taking and self-reported altruism subscales and infants’ sad and happy pupil dilation difference scores. We found that parental perspective taking was significantly associated with infants’ sad pupil dilation difference scores, *r*(44) = 0.31, *p* = 0.04, 95% CI [0.02, 0.56], and marginally associated with infants’ happy pupil dilation difference scores, *r*(44) = 0.28, *p* = 0.06, 95% CI [-0.02, 0.53] (see **Figure 2**). In addition, we found that the relation between parents’ self-reported altruism and infants’ composite pupil dilation difference scores was primarily driven by infants’ happy pupil dilation difference scores, *r*(43) = 0.37, *p* = 0.01, 95% CI [0.08, 0.60] (see **Figure 3**), as the relation between parents’ self-reported altruism and infants’ sad pupil dilation difference scores was non-significant, *r*(43) = 0.20, *p* = 0.18.

## Discussion

The present study investigated whether variability in a precursor to mature empathy, namely infants’ arousal in response to others’ emotions, as indexed by pupillary changes to others’ emotional displays, is related to differences in parents’ empathic and prosocial dispositions. As an initial step toward this aim, we sought to ensure that our task was a reliable and accurate measure of infants’ arousal, by replicating results of previous studies that have found greater arousal toward emotional stimuli (i.e., stimuli with a positive or negative valence) relative to neutral stimuli (Geangu et al., 2011b; see also Partala and Surakka, 2003; Bradley et al., 2008). Consistent with past research, we found that observation of the sad and happy emotions elicited infants’ arousal significantly more than the neutral emotion; moreover, observation of the sad emotion elicited significantly more arousal than the happy emotion.

However, the primary aim of the study was to investigate relations between infants’ pupillary changes to others’ emotional displays and parents’ empathic and prosocial dispositions. We found that empathic perspective taking, which reflects parents’ tendency to adopt the perspectives of others in social situations, was associated with increases in infants’ pupil dilation in response to other infants’ happy and sad emotional expressions. In contrast, we found that affective dimensions of parental empathy, specifically empathic concern, were not associated with infants’ pupillary responses toward others’ emotions. These findings suggest that parents who more frequently take other people’s perspectives are more likely to have infants who exhibit greater degrees of arousal in response to others’ emotions. In addition, we found that parents’ frequency of self-reported altruistic behavior was related to infants’ arousal in response to others’ emotions, particularly happiness, such that parents who more frequently act altruistically toward others are more likely to have infants who exhibit greater arousal in response to others’ happy emotions.

These findings raise some interesting possibilities regarding why parents’ empathic and prosocial dispositions were related to infants’ arousal in response to others’ emotions. One possibility is that adopting another person’s perspective and becoming aroused in response to others’ emotions share certain characteristics (e.g., Bengtsson and Arvidsson, 2011; Missana et al., 2014). That is, at baseline, both perspective taking and arousal in response to others’ emotions requires one to register that another person is having a different experience than one’s own. Thus, the significant relation between parental perspective taking and infants’ arousal could reflect shared abilities to recognize the experiences of others, particularly when others’ experiences differ from one’s own. It is possible that these shared abilities arise because of individual differences in how parents socialize their infants. For example, parents who are high in perspective taking have been found to provide their children with increased opportunities to recognize others’ unique perspectives, including their differing emotional states, by highlighting and emphasizing these in their interactions (Soenens et al., 2007; Farrant et al., 2012; van Lissa et al., 2014). Accordingly, it is possible that parents who score high in perspective taking are more likely to mirror their infants’ and others’ emotional expressions, which could provide infants with the relevant experience needed to register and become aroused by another’s emotional state. Indeed, adults who report higher levels of empathic perspective taking are more likely to mimic the behavior and expressions of those around them (e.g., Chartrand and Bargh, 1999; Galinsky et al., 2005; Genschow et al., 2013); in turn, imitation of emotional expressions has been linked to increased empathic perspective taking (Stel and Vonk, 2009). Thus, the relation between parental perspective taking and infants’ arousal could also be explained by this disposition influencing how frequently parents engage in activities that serve to identify and reflect other people’s behaviors and emotions. Of course, it is also possible that the relation between infants’ arousal toward others’ emotions and parental perspective taking represents shared genetic tendencies between parents and infants, and/or an interaction between genetics and socialization. Future work should seek to clarify the exact nature of this relation.

We also found an association between parents’ self-reported altruism and infants’ arousal in response to others’ emotions, and particularly their arousal in response to another’s display of happiness. One possible reason for this relation is that helping others, and becoming aroused in response to others’ happiness, share similar characteristics. That is, a strong motivator for performing helpful acts is a drive to see others’ happiness that results from offering help (Aknin et al., 2013; Barasch et al., 2014; Dunn et al., 2014; see also Telle and Pfister, 2012). For example, being able to see one’s recipient, as opposed to giving to others remotely, is associated with increased rates of charitable giving (Aknin et al., 2013). This suggests that seeing another person’s happiness, and knowing that one’s actions are the impetus for that emotion, is a strong motivator for altruistic behavior. In addition, it is possible that there are individual differences in how aroused adults are toward others’ displays of happiness (Heller, 1993). Thus, the selective association between infants’ arousal in response to others’ happiness and parental altruistic behavior may reflect a shared tendency to become aroused or motivated by another’s happiness. Similarly, and in support of this idea, Hepach et al. (2013) found that 2-year-old children’s pupil dilation predicted the speed with which children offered to help another person in need, such that greater pupil dilation was associated with quicker offers to help (Hepach et al., 2013; see also Hepach et al., 2012). Importantly, children in this study were not explicitly rewarded or recognized for offering to help the other person, which suggests that children were otherwise motivated, presumably by a desire to elicit and witness another person’s happiness. However, another, non-mutually exclusive possibility for this relation, given that helpful acts are likely to elicit happy emotions in both the helper and the helped, is that parents who perform more helpful acts toward others provide their infants with increased experience seeing other people’s happiness. As the frequency of observing emotions is linked to one’s ability to recognize and process them (Calvo et al., 2014), it is likely that infants with increased experience observing others’ happiness are better able to register and become aroused by others’ happiness displays. Thus, either a shared sensitivity toward others’ happiness, or differences in exposure to others’ happy emotional displays, may account for the relation between parents’ self-reported altruism and infants’ arousal toward others’ happiness in the present study.

Intriguingly, we did not find an association between parents’ empathic concern and infants’ arousal toward others’ emotions. This null relation is interesting in part because empathic concern assesses one’s other-oriented affective response toward another person’s distress, which ostensively bears similarity to infants’ arousal in response to others’ emotions. One possibility for this null relation is methodological: self-report measures of empathy, and particularly measures of empathic concern (see Einolf, 2008, for a review), are known to elicit socially desirable responses (Watson and Morris, 1991; Litvack-Miller et al., 1997; Zaki, 2014; see also Eisenberg and Miller, 1987), which would hamper demonstrating associations with infants’ arousal toward others’ emotions. Another possibility is that parents exhibit relatively homogenous, and high, levels of empathic concern in their real-life behavior toward their young infants, which is in contrast to what they report exhibiting in everyday life on questionnaire measures. In other words, given that infants are such compellingly helpless and adorable individuals, most anyone would be expected to exhibit high levels of empathic concern toward them, even those who ordinarily demonstrate low levels of empathic concern toward others. If this is the case, questionnaire measures may not accurately assess the level of empathic concern that parents demonstrate toward their infants, which would account for the lack of relation between self-reports of dispositional empathic concern and variability in infants’ arousal to others’ emotions in the present study. Future work may seek to further explore these possibilities.

Another issue that bears consideration is the meaning of infants’ arousal in response to others’ emotions, or what infants’ arousal in response to others’ emotions reflects. One possibility is that pupil dilation in response to others’ emotions reflects infants’ own feelings of personal distress. However, we believe that this is unlikely for several reasons. First, infants showed arousal in response to others’ expressions of both happiness and sadness, the former of which would not be expected to elicit distress. Second, parental personal distress showed a marginal negative relation with infants’ arousal toward others’ emotions, which indicates that parents with higher levels of personal distress had infants who exhibited less arousal in response to others’ emotional displays, which is the opposite of what would be expected if infants’ pupil dilation reflected personal distress. Lastly, no infants cried during observation of the videos, even the sad, and crying is commonly operationalized as reflecting personal distress in studies of early empathy (e.g., Roth-Hanania et al., 2011). Altogether, this demonstrates that infants registered and were subsequently aroused by the other infants’ emotional displays, without becoming upset by them. Indeed, in contrast to personal distress, we propose that infants’ arousal in response to others’ emotions reflects infants’ emerging sense of emotional attunement with others, or their sense of connectedness and responsivity to others’ emotions (see Markova and Legerstee, 2006). Indeed, emotional attunement is thought to be related to empathy (Gallese et al., 2007), which further suggests that infants’ arousal in response to others’ emotions reflects emotional attunement as opposed to personal distress.

More broadly, this study fits nicely into the literature on the development of empathy and earlier emerging precursors in young children. Specifically, this study compliments past work on infants’ arousal in response to others’ emotions by confirming that infants exhibit heightened and differential arousal toward others’ emotions (i.e., happiness and sadness) by the end of the first year of life (Geangu et al., 2011b). In addition, this study extends upon prior work that has investigated precursors to empathic responding (e.g., Sagi and Hoffman, 1976; Martin and Clark, 1982; Haviland and Lelwica, 1987; Termine and Izard, 1988; Dondi et al., 1999) by demonstrating that variability in infants’ arousal toward others’ emotions is accounted for by differences in their parents’ empathic and prosocial dispositions. This is important, as it provides further evidence that a precursor to empathic responding is meaningfully connected to mature empathy and theoretically aligned behaviors (Roth-Hanania et al., 2011). Accordingly, the present study encourages continued investigation into relations between variability in precursors to empathy and variability in fully developed empathic responding, in an effort to better understand its developmental trajectory; moreover, the present design provides a methodology for doing so. For example, an interesting question for future research would be to investigate whether variability in infants’ arousal in response to others’ emotions, as indexed by pupil dilation, is predictive of empathic dispositions in early childhood. In addition, this study calls for more work investigating parental dispositions as a source of variability in children’s early empathic responses. For example, future work may seek to investigate how heritability and socialization contribute to the relation between parental dispositions and infants’ arousal toward others’ emotions.

## Conclusion

This study supports investigating individual variability in infants’ early empathic responses as a phenomena of interest, rather than treating such variability as noise. Our results suggest that individual variability in infants’ arousal toward others’ emotions indexes meaningful differences in the manner in which infants are processing the social world. In addition, the present study confirms and extends upon prior work that has found a strong association between parental behaviors and their children’s developing empathy, by demonstrating that parents, construed as individuals, and not just in their capacity as parents *per se*, are significant predictors of their infants’ automatic responses to another’s emotional state. Altogether, the present study highlights the merits of using pupil dilation in response to others’ emotions as a measure of children’s emerging empathy and encourages directly investigating parental dispositions as a source of variability in these early empathic responses.
